# Factors associated with women’s preferences for labor epidural analgesia in Singapore: a survey approach

**DOI:** 10.1038/s41598-022-15152-3

**Published:** 2022-06-29

**Authors:** Chin Wen Tan, Semra Ozdemir, Rehena Sultana, Claire Tan, Hon Sen Tan, Ban Leong Sng

**Affiliations:** 1 Department of Women’s Anesthesia, KK Women’s and Children’s Hospital, Singapore, Singapore; 2 Anesthesiology and Perioperative Sciences Academic Clinical Program, Duke-NUS Medical School, Singapore, Singapore; 3 Lien Centre for Palliative Care, Duke-NUS Medical School, Singapore, Singapore; 4 Health Services and System Research, Duke-NUS Medical School, Singapore, Singapore; 5 Saw Swee Hock School of Public Health, National University of Singapore, Singapore, Singapore; 6 Centre for Quantitative Medicine, Duke-NUS Medical School, Singapore, Singapore; 7 Yong Loo Lin School of Medicine, National University of Singapore, Singapore, Singapore

**Keywords:** Health care, Human behaviour

## Abstract

Epidural analgesia provides effective pain relief during labor. However, there is limited information on the factors associated with pregnant women’s preferences for labor epidural analgesia (LEA) prior to labor onset. We performed a secondary analysis of a clinical trial to identify demographic characteristics, pain and psychological vulnerability factors associated with preferences for LEA. Pregnant women at ≥ 36 weeks’ gestation prior to labor and delivery were recruited and given questionnaires on their LEA preferences, psychological and pain vulnerabilities. The primary outcome was the association between pre-delivery Edinburgh Postnatal Depression Scale (EPDS) with cut-off ≥ 10 and LEA preference. Of the 250 women recruited, 51.6% (n = 129) indicated “yes to LEA”. Amongst those considering LEA as an option to reduce labor pain, women who preferred to use LEA (n = 129) indicated favorable or neutral opinion. Additionally, 68% (n = 82) from those “no to LEA” or “not sure about LEA” still gave either favorable or neutral opinion for LEA (*p* < 0.0001). The multivariate logistic regression analysis found that EPDS ≥ 10 (*p* < 0.01), occupation (*p* = 0.03), ethnicity (*p* < 0.01), state anxiety (*p* = 0.02), mode of current pregnancy (unplanned; planned, assisted; planned, natural; *p* = 0.03) and premenstrual anger/irritability before current pregnancy (*p* = 0.02) were associated with LEA preference. The findings may help to define the population that may require further education on considering LEA and allow early identification on different LEA preferences to provide patient centric care prior to labor and delivery.

## Introduction

Childbirth pain is one of the most excruciating types of pain a woman might have to endure in her lifetime and pain management is an essential component of labor and delivery^1^. Labor epidural analgesia (LEA) is considered the reference standard for pain relief, and is recommended by the World Health Organization for healthy laboring women^2, 3^. Additionally, LEA may benefit women with high-risk pregnancies such as preeclampsia, owing to its ability to reduce pain-related hypertension and catecholamine levels while potentially improving uteroplacental flow^4^. However, despite the advantages of LEA, many women expressed concerns over its potential adverse effects including prolonged labor, need for instrumental delivery, and back pain^5,6^. Furthermore, a qualitative systematic review investigating labor experiences showed that women who received unplanned LEA were more likely to experience negative emotions such as guilt, conflict, and sense of failure^7^. As a whole, these studies highlight the complex and multifaceted aspects regarding maternal acceptance of LEA and may partially explain the discrepancy in LEA uptake between our study center (~ 50%)^8^ compared to other developed countries such as the United States (~ 71%) and Canada (~ 59%)^9,10^. Hence, in-depth understanding of factors affecting LEA preference is needed to define the population that may benefit from additional counselling or education, with the goal of providing pertinent and relevant information required to make an informed decision regarding LEA^11^.

Although several studies have reported associations between maternal socioeconomic (e.g., education, income, parity) and psychological factors (e.g., stress, anxiety, fear) with LEA preferences^12–14^, the relationship between other psychological and pain vulnerability factors (e.g., depressive symptoms, pain catastrophizing etc.) and LEA acceptance is unclear. For instance, previous studies on pre-delivery depressive symptoms reported conflicting results: Sitras et al. found no significant difference in Edinburgh Postnatal Depression Scale (EPDS) scores among women who had chosen or not chosen LEA, whilst Chung et al. showed that increased Beck Depression Inventory (BDI) scores at late pregnancy were associated with increased LEA utilization^15,16^. Nonetheless, both studies were conducted in women at around 32 gestational weeks, and their findings may not reflect actual LEA preference nearer to term.

In this study, we aimed to identify pre-delivery factors including the presence of depressive symptoms (primary exposure variable), socioeconomic characteristics, and pain and psychological vulnerability factors (secondary exposure variables) associated with preference for LEA (primary outcome measure). We hypothesized that the binary variable of pre-delivery EPDS score ≥ 10 is associated with preference for LEA.

## Methods

This study is a secondary analysis of a larger randomized-controlled study investigating the association between LEA and postpartum depression, conducted at KK Women’s and Children’s Hospital, the largest specialist obstetric hospital in Singapore. Enrolment for the primary study has been completed, but participant follow-up and data analysis are still ongoing. Both the primary study and our secondary analysis were approved by SingHealth Centralized Institutional Review Board (reference number: 2017/2090; registered date: 25 Mar 2017) and registered on Clinicaltrials.gov (NCT03167905) (initial release: 2 May 2017; first posted: 30 May 2017). Eligible women enrolled from June 2018 to November 2020 were approached to participate, and written informed consent was obtained from all participants. This manuscript adheres to the applicable Strengthening the Reporting of Observational Studies in Epidemiology (STROBE) guidelines.

We included pregnant women aged 21–50 years old with American Society of Anesthesiologists (ASA) physical status II, nulliparous or multiparous, with a singleton fetus of ≥ 36 gestational weeks. Women with non-cephalic fetal presentation, obstetric complications, uncontrolled medical conditions (e.g., cardiac disease), and who underwent elective caesarean delivery were excluded. To enable the use of standardized validated English questionnaires, participants who were unable to understand or read English were excluded.

Participants would receive a questionnaire on their preferences for labor analgesia commonly used in our institution (LEA, pethidine, or 50% nitrous oxide/oxygen (Entonox)), opinions on LEA and its potential adverse effects (prolonged labor, increased risk of instrumental delivery, back pain, permanent nerve injury, and neonatal respiratory respiration) (Supplementary information). Information on adverse effects of non-LEA interventions were also assessed in the questionnaire. We pre-tested the first draft of the questionnaire in ten eligible participants to determine if they were able to understand the questions, and whether all their concerns on labor epidural analgesia were captured. We then refined the questionnaire based on provided feedback. In addition, the following validated questionnaires on pain and psychological vulnerability were administered 30–40 min prior to labor and delivery:

(i) Edinburgh Postnatal Depression Scale (EPDS): A 10-item self-reporting scale to screen for antenatal and postnatal depression (PND), with a score ranging from 0 to 30^17,18^. EPDS is moderately correlated with BDI (Spearman correlation: 0.78, *p* < 0.001)^19^, and has been well validated in both antenatal and postnatal women population in Singapore^20^;
(ii) Pain Catastrophizing Scale (PCS): a self-reported instrument on evaluating negative thought processes that one may have upon exposure to actual or perceived pain and/or painful experiences^21^;
(iii) Central Sensitization Inventory (CSI): a psychometric instrument that evaluates one’s responses to expansion of pain, and prolonged pain once the stimulus is removed^22^;
(iv) Perceived Stress Scale (PSS): an instrument commonly used for quantifying perceptions of stress^23^;
(v) Fear-Avoidance Components Scale (FACS): a patient-reported measure that serves to evaluate the fear-avoidance in patients especially those with painful conditions^24^;
(vi) State-Trait Anxiety Inventory (STAI): a 40-item instrument to assess transient anxiety (STAI State anxiety), dispositional anxiety (STAI Trait anxiety), and anxiety in general (STAI total)^25^. A cut-off of 40 was generally used to identify elevated anxiety during pregnancy^26^;
(vii) Premenstrual Symptoms Screening Tool (PSST): a self-reported screening tool to identify women with severe Premenstrual Syndrome (PMS) or Premenstrual Dysphoric Disorder (PMDD)^27^. As the purpose of the study was to look at the relationship between psychological characteristics and LEA preference, we decided to analyze the individual items of PSST, rather than investigating the premenstrual disorders of women;
(viii) EQ-5D-3L: a widely used instrument to quantify health-related quality of life^28^.

Patient characteristics and socioeconomic information including body mass index, marital status, housing type (private or public), housing status (rented or owned), highest education, occupation, children, mode of current pregnancy (unplanned; planned, assisted; planned, natural) and gestational age were also collected.

The primary objective was the association between EPDS ≥ 10 and LEA preference. LEA preference was treated as categorical data: “yes to LEA” for participants who chose LEA; “no to LEA” for those who chose Entonox, pethidine, or who did not want any analgesia; and “not sure about LEA” for those who were undecided regarding their labor analgesia. EPDS ≥ 10 indicates the presence of clinically significant depressive symptoms^17,18,29^. Categorical and continuous variables were summarized as frequency (proportion), mean (standard deviation (SD)) based on LEA preference. Differences between the outcome categories were tested using analysis of variance (ANOVA) for continuous variables and using Chi-square test for categorical variables. Univariate and multivariable multinomial logistic regression were performed to investigate the factors associated with LEA preference, and expressed as odds ratios (OR) with corresponding 95% confidence intervals (CI). Variables with *p* < 0.20 in the univariate logistic regression analysis were chosen for multivariable logistic regression model, which was subsequently finalized using forward, backward, and stepwise variable selection methods. Significance was set at *p* < 0.05 and all tests were two-tailed. No correction for multiple comparisons was made. SAS version 9.4 software (SAS Institute; Cary, North Carolina, USA) was used in all analyses.

## Results

**Figure 1:**
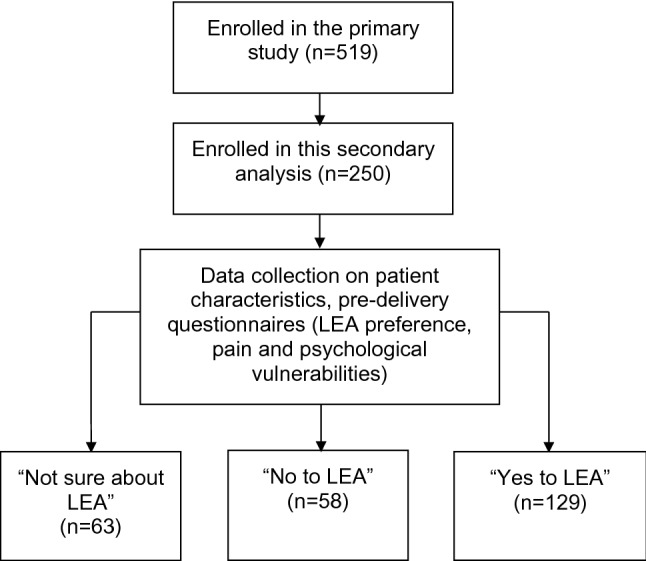
Study flowchart. LEA, labor epidural analgesia.

We enrolled 250 participants prior to labor and delivery, categorized according to their preferences for LEA (“yes to LEA”: n = 129; “no to LEA”: n = 58; and “not sure about LEA”: n = 63) (Fig. 1). Participant characteristics and socioeconomic information are summarized in Table 1, with ethnicity, occupation, age, and mode of current pregnancy significantly associated with LEA preference based on univariate analysis.

**Table 1:** Demographic characteristics.

Characteristics	Not sure about LEA N = 63	No to LEA N = 58	Yes to LEA N = 129	*p *value
Age (years), mean (SD)	30.3 (3.8)	29.9 (3.7)	31.7 (3.4)	< 0.005
Ethnicity, *n* (%)				< 0.001
Chinese	33 (52.4)	22 (38.0)	92 (71.9)	
Malay	19 (30.2)	28 (48.3)	22 (17.2)	
Indian	3 (4.8)	2 (3.4)	5 (3.9)	
Others	8 (12.6)	6 (10.3)	9 (7.0)	
Weight (kg), mean (SD)	75.0 (16.1)	72.9 (14.0)	71.4 (11.3)	0.32
BMI (kg/m^2^), mean (SD)	29.1 (5.2)	29.5 (6.5)	27.9 (4.2)	0.53
Marital status, *n* (%)				0.75
Single	1 (1.6)	2 (3.4)	3 (2.3)	
Married	62 (98.4)	56 (96.6)	126 (97.7)	
Children, *n* (%)				0.10
No	50 (82.0)	37 (64.9)	92 (71.3)	
Yes	11 (18.0)	20 (35.1)	37 (28.7)	
Current pregnancy, *n* (%)				< 0.01
Unplanned	25 (40.3)	17 (30.4)	20 (15.7)	
Planned, natural	32 (51.6)	34 (60.7)	91 (71.7)	
Planned, assisted	5 (8.1)	5 (8.9)	16 (12.6)	
Gestational age (weeks), mean (SD)	39.2 (1.0)	39.2 (1.0)	39.2 (0.9)	0.91
Highest education, *n* (%)				0.91
Post-secondary and below	9 (14.5)	8 (14.8)	15 (12.5)	
Post-graduate	53 (85.5)	46 (85.2)	105 (87.5)	
Occupation, *n* (%)				0.02
Homemaker/unemployed	30 (49.2)	24 (44.5)	40 (31.7)	
Self-employed	8 (13.1)	10 (18.5)	12 (9.6)	
Professional	23 (37.7)	20 (37.0)	74 (58.7)	
Housing type, *n* (%)				0.64
Public	52 (91.2)	42 (87.5)	103 (92.0)	
Private	5 (8.8)	6 (12.5)	9 (8.0)	
Housing status, *n* (%)				0.47
Rented	6 (11.3)	7 (16.3)	10 (9.5)	
Owned	47 (88.7)	36 (83.7)	95 (90.5)	

The missing data on demographic characteristics are as follows: Weight (n = 84), BMI (n = 85), housing type (n = 33), and housing status (n = 49).

*BMI* body mass index; *LEA* labor epidural analgesia.

When asked about their view on LEA as an option to reduce labor pain, all participants answering “yes to LEA” (n = 129) also responded favorably or neutrally (Table 2). In addition, 68% (n = 82) of participants answering “no to LEA” or “not sure about LEA” still gave either favorable or neutral opinions regarding LEA as an option for labor pain relief. Also, significant differences were found between participants answering “yes to LEA”, “no to LEA” and “not sure about LEA” regarding the perception that the second stage of labor will last one hour or more and LEA increases the risk of adverse events (permanent nerve injury, neonatal respiratory depression).

**Table 2:** LEA preferences and other opinions on labor and delivery.

Characteristics	Not sure about LEA N = 63	No to LEA N = 58	Yes to LEA N = 129	*p *value
Planned labor analgesia (multiple selection was allowed), *n* (%)				
Epidural analgesia	–	–	129 (100.0)	–
Pethidine	0	5 (8.6)	6 (4.7)	< 0.05
Entonox	1 (1.6)	45 (77.6)	27 (20.9)	< 0.0001
Not sure	63 (100.0)	–	–	< 0.0001
Do not want any analgesia	–	11 (19.0)	–	< 0.0001
Positive or neutral opinion of epidural analgesia as an option for analgesia, *n* (%)	48 (76.2)	34 (58.6)	129 (100.0)	< 0.0001
Perceived second stage of labor would last ≥ one hour, *n* (%)	26 (51.0)	23 (44.2)	81 (73.6)	< 0.001
Perceived longer second stage of labor if epidural analgesia is used, *n* (%)	39 (76.5)	33 (67.3)	77 (65.8)	0.38
Perceived higher chance of getting instrumental delivery if epidural analgesia is used, *n* (%)	35 (72.9)	44 (86.3)	79 (72.5)	0.14
Perceived higher chance of getting back pain if epidural analgesia is used, *n* (%)	52 (91.2)	47 (88.7)	101 (87.1)	0.72
Perceived higher chance of getting permanent nerve injury if epidural analgesia is used, *n* (%)	51 (81.0)	53 (91.4)	85 (65.9)	< 0.001
Perceived higher chance of getting neonatal respiratory depression if epidural analgesia is used, *n* (%)	54 (85.7)	48 (82.8)	86 (66.7)	< 0.01

*LEA* labor epidural analgesia.

Pre-delivery pain and psychological vulnerabilities in the recruited women, including central sensitization (CSI), depressive symptoms (EPDS), global health score (EQ-5D-3L), fear-avoidance (FACS), pain catastrophizing (PCS), perceived stress (PSS), and premenstrual symptoms before their current pregnancy (PSST) are shown in Table 3. The multivariable logistic regression analysis found that EPDS ≥ 10, occupation, ethnicity, state anxiety, mode of current pregnancy, and having premenstrual anger/irritability before the current pregnancy were significantly associated with LEA preference (Table 4).

**Table 3:** Pain and psychological vulnerabilities.

Parameters	Not sure about LEA N = 63	No to LEA N = 58	Yes to LEA N = 129	*p *value
CSI (0–100), mean (SD)	38.8 (16.1)	37.8 (19.4)	40.4 (17.1)	0.84
EPDS (0–30), mean (SD)	7.6 (3.6)	7.6 (4.2)	7.9 (4.4)	0.86
EPDS ≥ 10, *n* (%)				0.05
No	59 (95.2)	50 (86.2)	107 (82.9)	
Yes	3 (4.8)	8 (13.8)	22 (17.1)	
EQ-5D-3L global health score (0–100), mean (SD)	73.0 (17.4)	72.0 (16.8)	76.3 (13.9)	0.15
FACS (0–100), mean (SD)	38.8 (16.1)	37.8 (19.4)	40.4 (17.1)	0.61
PCS- Rumination (0–16), mean (SD)	8.0 (4.0)	7.1 (4.5)	7.3 (4.1)	0.47
PCS- Magnification (0–12), mean (SD)	4.4 (3.0)	4.1 (2.9)	4.0 (2.6)	0.70
PCS- Helplessness (0–24), mean (SD)	8.6 (5.5)	7.6 (5.3)	7.5 (4.8)	0.39
PCS- Total Score (0–52), mean (SD)	20.9 (11.4)	18.8 (11.7)	18.9 (10.3)	0.43
PSS (0–40), mean (SD)	12.8 (4.3)	13.0 (4.9)	12.8 (4.4)	0.95
PSST item 1: Anger/irritability, *n* (%)				0.02
Not at all	10 (16.1)	21 (36.2)	26 (20.2)	
Mild/moderate/severe	52 (83.9)	37 (63.8)	103 (79.8)	
STAI- State anxiety (20–80), mean (SD)	41.4 (10.4)	38.5 (11.8)	38.2 (11.6)	0.18
STAI- State anxiety ≥ 40, *n* (%)				0.37
No	31 (49.2)	35 (60.3)	76 (58.9)	
Yes	32 (50.8)	23 (39.7)	53 (41.1)	
STAI- Trait anxiety (20–80), mean (SD)	38.9 (8.6)	38.6 (9.8)	37.8 (9.3)	0.73
STAI- Trait anxiety ≥ 40, *n* (%)				0.72
No	39 (61.9)	39 (67.2)	79 (61.2)	
Yes	24 (38.1)	19 (32.8)	50 (38.8)	
STAI- Total Score (40–160), mean (SD)	80.2 (17.3)	77.1 (20.5)	76.0 (19.6)	0.36

The missing data on questionnaires are as follows: CSI (n = 2), EPDS (n = 1), EQ-5D-3L (n = 1), FACS (n = 7), PCS (n = 2), PSS (n = 1), PSST (n = 1), and STAI (n = 1).

*CI* confidence interval, *CSI* central sensitisation inventory, *EPDS* Edinburgh postnatal depression scale, *EQ-5D-3L* EuroQol five-dimensional-three-level, *FACS* fear-avoidance components scale, *LEA* labor epidural analgesia, *OR* odds ratio, *PCS* pain catastrophizing scale, *PSS* perceived stress scale, *PSST* premenstrual symptoms screening tool, *STAI* state-trait anxiety inventory.

**Table 4:** Multivariable logistics regression for LEA preference.

Variable	No to LEA		Not sure about LEA		Overall *p* value
	Adjusted OR (95% CI)	*p* value	Adjusted OR (95% CI)	*p* value	
EPDS ≥ 10	0.47 (0.15–1.49)	0.20	0.11 (0.03–0.45)	< 0.005	< 0.01
Occupation					0.03
Professional (Ref: Homemaker/unemployed)	0.41 (0.19–0.90)	0.03	0.45 (0.22–0.95)	0.04	
Self-employed (Ref: Homemaker/unemployed)	1.55 (0.53–4.55)	0.43	1.24 (0.42–3.67)	0.70	
Ethnicity					< 0.01
Indian (Ref: Chinese)	1.74 (0.29–10.58)	0.55	0.62 (0.10–3.84)	0.61	
Malay (Ref: Chinese)	4.75 (2.16–10.46)	< 0.001	1.85 (0.83–4.13)	0.13	
Others (Ref: Chinese)	2.94 (0.88–9.86)	0.08	2.21(0.72–6.76)	0.17	
STAI- State anxiety	1.01 (0.98–1.05)	0.45	1.05 (1.01–1.08)	< 0.01	0.02
Current pregnancy					0.03
Planned, assisted (Ref: Planned, natural)	0.92 (0.29–2.94)	0.89	0.86 (0.28–2.67)	0.79	
Unplanned (Ref: Planned, natural)	1.92 (0.83–4.46)	0.13	3.58 (1.61–7.97)	< 0.005	
PSST item 1: Anger/irritability	0.35 (0.15–0.79)	0.01	0.98 (0.39–2.44)	0.97	0.02

Adjusted ORs were obtained from multivariable logistic regression by taking potential confounders (*p* < 0.20) identified by univariate analysis. The reference group is “yes to LEA”.

*CI* confidence interval, *EPDS* Edinburgh postnatal depression scale, *LEA* labor epidural analgesia, *OR* odds ratio, *PSST* premenstrual symptoms screening tool, *STAI* state-trait anxiety inventory.

Comparing women answering “yes to LEA” versus “no to LEA”, those with a professional job and having premenstrual anger/irritability before the current pregnancy were more likely to choose LEA as their labor analgesia (Table 4). Conversely, Malay ethnic group, as compared with Chinese, were less likely to choose LEA for pain relief. Similarly, those having an EPDS ≥ 10, having a professional job, less state anxiety, and having a planned/natural current pregnancy were more likely to choose LEA as compared with those who were unsure about their LEA preference.

## Discussion

In this study, we evaluated women’s preferences for LEA using a survey approach. Pregnant women devised their preference on LEA mainly in three different categories: Yes, no, or not sure. In the multivariable analysis, we found that pre-delivery depressive symptoms (EPDS ≥ 10), occupation, ethnicity, state anxiety, mode of current pregnancy and having premenstrual anger/irritability before current pregnancy were significantly associated with LEA preference.

To date, few studies have investigated factors affecting maternal LEA preferences, particularly regarding pain, mood, and other feelings prior to onset of labor. This study underscores the association between psychological factors and LEA preference. Previous studies have investigated the influence of depressive symptoms on LEA preference^15,16^. In our study, we showed that a significant association was found between LEA preference and pre-delivery depressive symptoms as indicated by an EPDS cut-off of 10 for minor depressed conditions^30^; but this effect was dominated by a significant positive association between EPDS ≥ 10 and “yes to LEA” as compared with those who were unsure about their LEA preference. Similarly, we showed a significant association between state anxiety (transitory emotional state) and LEA preference in multivariate analysis. One possible theory is that the negative psychological effects, be it from anxiety or depression, may magnify one’s pain perception or lower the pain threshold, which in turn amplifies both physical and psychological distress^31^, however further affirmation is required.

Premenstrual symptoms vary among individuals, including significant anger/ irritability, anxiety/tension, and somatic complaints. A history of premenstrual syndrome (PMS) has been found to be associated with the development of postnatal depression (PND) at later time. A systematic review on 16 studies showed a positive association between premenstrual disorders and PND, and this relationship may be modulated by confounders on vulnerable personality and socio-economic status e.g., age, income, and education^32^. It has been speculated that vulnerability to hormonal alterations during reproductive years may contribute not only to the development of PMS but also of PND, thus leading to postnatal depressive and other mood symptoms^33,34^. To our knowledge, no study has investigated the association between premenstrual syndromes and LEA preference, and our results showed that premenstrual anger/irritability was the only domain in PSST that was associated with LEA preference. Notably, data on relationship between premenstrual symptoms PND are usually collected retrospectively during pregnancy, this reporting may be influenced by concurrent anxiety or depressive symptoms before delivery, and no further hormonal assessment was performed to confirm the association. Given that pre-labor period involves dramatic changes in endocrine events, it would be interesting to further investigate the difference in hormonal changes among those preferred or not preferred LEA.

As part of the survey, we investigated various demographic and socioeconomic factors that may affect women’s LEA preference. In the current study, working as a professional as compared with homemaker and unemployed mothers was a significant predictor of positive LEA preference. This is consistent with previous reported studies, whereby employed mothers are more likely to choose LEA than non-employed women^35,36^. Notably, the study by Le Ray et al. was conducted in France, a country with high rate of LEA use (77%) with epidural procedures 100 percent reimbursed via health insurance; hence the contributing factor of employment is not confounded by the affordability of obstetric care^36,37^. In Singapore, the delivery charges including the epidural administration are payable by the pregnant women. However, a “Medisave Maternity Package” which is part of the national medical savings scheme could allow pregnant women to claim up to $3150 regardless of the ward type they have chosen. The amount could fully cover the delivery expenses for subsidized patients, and up to 60% for unsubsidized patients choosing single-bed or 4-bed wards^38–40^. As education level does not show significance in univariate analysis, it is plausible that those who works as a professional may receive more resources for adequate prenatal care, yet this warrants further study^36^. Reports of LEA preference in Asian population were nonetheless conflicting: Harkins et al. showed that there was no difference in LEA preference in East Asian and Indian Asian, probably attributed to a small sample size, whereas Sharma et al. showed that Malay mothers were less likely to consent to LEA as compared with non-Malay ethnic groups (Chinese, Indian etc.), which is in line with our findings^6,41^. This could be possibly explained by cultural and religious reasons for LEA refusal, where traditional belief regards pain as a necessary experience of childbirth^41^.

Apart from LEA preference, we also collected information on women’s general perception on the duration of second stage of labor, and showed that among the three groups of different LEA preference, those who were more likely to choose LEA expected themselves laboring for a longer time. We hypothesized that women who chose LEA might be more anxious about their upcoming labor, however by conducting a post hoc analysis on the association between LEA preference and STAI score, we found that the correlation was weak (R = 0.10), suggesting that there are other possible confounders that may have determined the perception on the duration of second stage of labor. Additionally, we also found significant difference on how women perceived the risk of adverse events if epidural analgesia was used, in particular permanent nerve injury and neonatal respiratory depression. This finding is similar to Harkins et al. where a significant portion of those who did not obtain an epidural analgesia stated the concern of possible risks to them or the babies (~ 28%)^6^.

We evaluated how women’s preferences on LEA differ among individuals and presented a view on their perception on LEA versus non-LEA pain relief options. Munro et al. found that women preferred to receive relevant information during prenatal period rather than during labor. Moreover, patients prefer receiving information provided by their primary-care provider, which they trusted more, rather than an anesthesiologist they do not know^12^. Information received during labor could be limited, and pregnant women may find it challenging to learn and understand about the risks prior to decision-making^42^. This study suggests that perhaps a more personalized approach should be taken, of which the identification of the associated demographic and psychological factors may serve to stratify women of different needs for labor pain relief, especially in those who are not sure or those who do not want LEA, so that dedicated information could be provided to ease their concerns on the use of LEA.

However, this study has several limitations. First, we recruited women from single maternity institution with a predominantly Asian population. It was previously reported that Asians may have different perceptions towards pain as compared with other populations of different cultural and demographic context, hence limiting the generalizability of the study^43^. We have found several factors that may contribute to LEA preference; however, a larger sample size is needed to validate the findings. In addition, the present study is a secondary analysis and hence no sample size calculation was performed. Post hoc power calculation is not suitable for this objective as the post hoc power estimates is different from true power calculation which may not provide sensible results. Secondly, the recruitment was limited to English-speaking women and potential confounding effects (e.g., previous childbirth/ LEA experiences, household or personal income, information source and quality received before labor) that may affect women’s perceptions were not studied. It is also notable that the stated LEA preferences prior to their labor and delivery may not reflect their actual choices, which can be affected by emotional, financial, and clinical situation not considered in the survey. Women may also consider the preferences of their spouse or other family members when making treatment choices.

In summary, our survey reflects that pre-delivery demographic and psychological characteristics are associated with LEA preference prior to delivery and labor. Future studies could help to define the population that may require further education on considering LEA, including how benefits and risks associated with epidural analgesia could impact the LEA preference and the actual use of different analgesia modalities. Early identification on women’s different perception on LEA preference based on the associated factors will also enable healthcare professionals to provide patient centric care to improve women’s labor experience.

## Supplementary Information

Supplementary Information.

## Data Availability

The datasets generated and analyzed in this work are available for anyone who wishes to access the data by contacting the corresponding author.

## Abbreviations

ASA: American society of anesthesiologists
BDI: Beck depression inventory
BMI: Body mass index
CI: Confidence interval
CSI: Central sensitisation inventory
EPDS: Edinburgh postnatal depression scale
EQ-5D-3L: EuroQol five-dimensional-three-level
FACS: Fear-avoidance components scale
LEA: Labor epidural analgesia
OR: Odds ratio
PCS: Pain catastrophizing scale
PMDD: Premenstrual dysphoric disorder
PMS: Premenstrual syndrome
PND: Postnatal depression
PSS: Perceived stress scale
PSST: Premenstrual symptoms screening tool
STAI: State-trait anxiety inventory
STROBE: Strengthening the reporting of observational studies in epidemiology
