# Supplementary material for: Factors associated with women’s preferences for labor epidural analgesia in Singapore: a survey approach
Source: Sci Rep. 2022 Jun 29;12:10961. doi: 10.1038/s41598-022-15152-3 (PMC9242983; doi:10.1038/s41598-022-15152-3)
Supplement: Supplementary file 1 — Supplementary Information. [file 41598_2022_15152_MOESM1_ESM.pdf]

Subject Number: _______ **Date of Completion: ___ / ___ / ___ (DD / MM / YY)**

Subject Ward:__________

PREFERENCES FOR LABOUR PAIN RELIEF- 1

This survey is about women's preferences for methods to reduce labour pain.

**A1.**	Which pain relief method have you decided to use to reduce your labour pain?<br><br>☐ Epidural<br>☐ Pethidine<br>☐ Laughing gas<br>☐ Not Sure/ Not decided yet<br>☐ I do not want to use a pain relief medicine

Type of pain relief method:

Epidural: Epidural provides pain relief in the lower part of the body while allowing you to remain conscious. A health care provider inserts a fine plastic tube into the epidural space of your spinal cord. Pain relief drug is delivered through this plastic tube via a pump. It takes 5 to 15 minutes for epidural to start relieving your pain and will provide you continuous pain relief.

**A2.**	**What is your opinion of <u>epidural</u> as an option to reduce labour pain?**<br><br>☐ Mostly positive<br>☐ Somewhat positive<br>☐ Neutral<br>☐ Somewhat negative<br>☐ Mostly negative<br>☐ Not sure<br>☐ Never heard of it

Pethidine: Pethidine is a pain relief medicine. Your nurse will inject the pethidine into your thigh or buttock and repeat the dose if you need it. Pethidine can make you feel sick, so the injection is likely to contain another drug to control sickness. It takes 20 to 30 minutes for pethidine to start working. Once the pethidine takes effect, it lasts for 2 to 4 hours.

**A3.**	**What is your opinion of <u>pethidine</u> as an option to reduce labour pain?**<br><br>☐ Mostly positive<br>☐ Somewhat positive<br>☐ Neutral<br>☐ Somewhat negative<br>☐ Mostly negative<br>☐ Not sure<br>☐ Never heard of it

Laughing gas: Laughing gas is a clear, colourless gas that is used for pain relief. You need to use a mask or mouth piece to inhale gas from a machine beside the bed. You need to take deep and steady breaths before each contraction as it takes about 20-30 seconds for the gas to be effective. When you breathe in, it leads to feelings of relaxation and detachment from your current situation. Laughing gas will not numb any parts of your body.

**A4.**	**What is your opinion of <u>laughing gas</u> as an option to reduce labour pain?**<br><br>☐ Mostly positive<br>☐ Somewhat positive<br>☐ Neutral<br>☐ Somewhat negative<br>☐ Mostly negative<br>☐ Not sure<br>☐ Never heard of it

Now we would like to know whether you had previous experience with giving birth.

**A5.**	**Have you given birth before?**<br><br>☐ Yes<br>☐ No
**A6.**	**[If A5 = Yes] Which pain relief method did you use in your previous labour?**<br><br>☐ Epidural<br>☐ Laughing gas<br>☐ Pethidine<br>☐ Other ________________________<br>☐ I did not use any pain relief<br>☐ I had a C-section
**A7.**	**[If used pain relief medicine] How satisfied were you with your pain relief method?**<br><br>Please mark on the scale below:<br><br>0 10 20 30 40 50 60 70 80 90 100<br>**Not at all** satisfied ... **Extremely** satisfied

Amount of pain after using pain relief medicine:

After using pain relief medicine, some women experience complete pain relief or only slight pain while other women may still have moderate to severe pain. The same pain relief method may work differently for different women.

**B1.**	**How much pain do you think women have during labour if they do not use any pain relief medicine?**<br><br>Please mark on the scale below:<br><br>0 1 2 3 4 5 6 7 8 9 10<br><br>**No** pain — **Worst** pain imaginable<br><br>☐ Not sure
**B2.**	**How about if they use an epidural?**<br><br>Please mark on the scale below:<br><br>0 1 2 3 4 5 6 7 8 9 10<br><br>**No** pain — **Worst** pain imaginable<br><br>☐ Not sure

Time taken for pushing the baby out:

The length of time it takes to push a baby out varies with the position and size of the baby and the woman's ability to push. It is possible that some pain relief methods may affect the urge to push and the length of time it takes to push the baby out.

**B3.**	**How long do you think it takes to push the baby out on average?**<br><br>☐ Less than half an hour<br>☐ Half an hour to 1 hour<br>☐ More than 1 hour but less than 2 hours<br>☐ More than 2 hour but less than 3 hours<br>☐ More than 3 hour but less than 4 hours<br>☐ More than 4 hour but less than 5 hours<br>☐ Not sure
**B4.**	**How about if women use an <u>epidural,</u> do you think it takes shorter or longer?**<br><br>☐ Much shorter<br>☐ Slightly shorter<br>☐ About the same<br>☐ Slightly longer<br>☐ Much longer<br>☐ Not sure

Number of women who have instrumental delivery

After receiving pain relief medicine, some women may lose the desire and ability to bear down and push. This may increase the chance of instrumental delivery, such as forceps and vacuum extractions.

**B5.**	**Compared to not using any pain relief medicine, do you think the chance of women having an instrumental delivery will be lower or higher <u>if they use an epidural?</u>**<br><br>☐ Much lower<br>☐ Slightly lower<br>☐ About the same<br>☐ Slightly higher<br>☐ Much higher<br>☐ Not sure

Number of women who have back pain after delivery

Some women experience back pain for several weeks after delivery regardless of using pain relief medicine.

**B6.**	**Compared to not using any pain relief medicine, do you think the chance of women having a back pain will be lower or higher <u>if they use an epidural?</u>**<br><br>☐ Much lower<br>☐ Slightly lower<br>☐ About the same<br>☐ Slightly higher<br>☐ Much higher<br>☐ Not sure

Number of women who have nerve injury due to epidural

In rare circumstances, some women may experience nerve injury due to epidural. The majority of women who experience lower limb weakness or numbness recover between a few days to a few weeks. Very rarely, nerve injury can be permanent and is associated with lower limb weakness, numbness and/or loss of control of the bowel or bladder.

**B7.**	**How worried are you about chance of having a permanent nerve injury due to epidural?**<br><br>☐ Very worried<br>☐ Somewhat worried<br>☐ Not worried<br>☐ Not sure

Number of babies who have breathing distress due to pain relief medicine

In rare cases, some pain relief medicine may cross the placenta to the baby to make him/her drowsy and the baby may experience slow or shallow breathing after he/she is born. If a baby experiences slow or shallow breathing, an "antidote" can be given to the baby to reverse these side effects.

**B8.**	**How worried are you about chance of your baby having breathing distress due to pain relief medicine?**<br><br>☐ Very worried<br>☐ Somewhat worried<br>☐ Not worried<br>☐ Not sure
